# A rapid streamline-based extension of Tractfinder for white matter tract segmentation

**DOI:** 10.3389/fnimg.2026.1873040

**Published:** 2026-07-06

**Authors:** Dana Kanel, Fiona Young, Kiran K. Seunarine, Chris A. Clark, Kristian Aquilina, Jonathan D. Clayden

**Affiliations:** 1 Developmental Imaging and Biophysics Section, UCL GOS Institute of Child Health, London, United Kingdom; 2 Software Engineering and Artificial Intelligence Science Technology Platform, The Francis Crick Institute, London, United Kingdom; 3 Department of Neurosurgery, Great Ormond Street Hospital for Children, London, United Kingdom

**Keywords:** dMRI, paediatric brain tumours, pre-operative, tract segmentation, tractography

## Abstract

Accurate delineation of white matter tracts is critical in the pre-operative assessment of paediatric brain tumour patients, where preservation of eloquent pathways directly influences surgical planning and functional outcomes. Tractfinder is a recently introduced automated method for white matter tract segmentation in tumour patients, but its voxel-based (mask) outputs limit compatibility with streamline-based tractography tools, visualisation workflows, and downstream analytical frameworks. Here we introduce Tractfinder-constrained Tractography (TcT), a streamline-based extension that constrains probabilistic tractography to the probability maps produced by Tractfinder, generating streamline representations while preserving the speed and automation that make Tractfinder clinically appealing. We evaluated TcT in ten pre-operative paediatric patients with supratentorial tumours, targeting three clinically relevant tracts – the corticospinal tract, arcuate fasciculus, and optic radiation. Spatial agreement between TcT and conventional tractography was assessed using Bundle Adjacency (BA). Mean BA scores across all three tracts ranged from 2.1 to 2.6 mm, comparing favourably against published inter-protocol benchmarks for conventional probabilistic tractography (4.3 mm), and approaching within-protocol variability. The TcT pipeline was fully automated, required no manual region-of-interest placement, and completed in approximately 5–15 min per subject compared to 1–2 h for conventional tractography. These results demonstrate that TcT produces streamline-based tract segmentations with good spatial agreement to conventional tractography, while offering substantially reduced processing time and operator burden.

## Introduction

Accurate delineation of white matter tracts is critical in the pre-operative assessment of brain tumour patients, where preservation of eloquent pathways directly influences surgical planning and functional outcomes. Tractfinder (Young et al., 2022, 2024) has recently been introduced as a rapid and practical method for outlining white matter tracts in tumour patients, generating binary masks of tract anatomy. Its principal strengths lie in its automation, speed, and ease of implementation.

**Figure 1:**
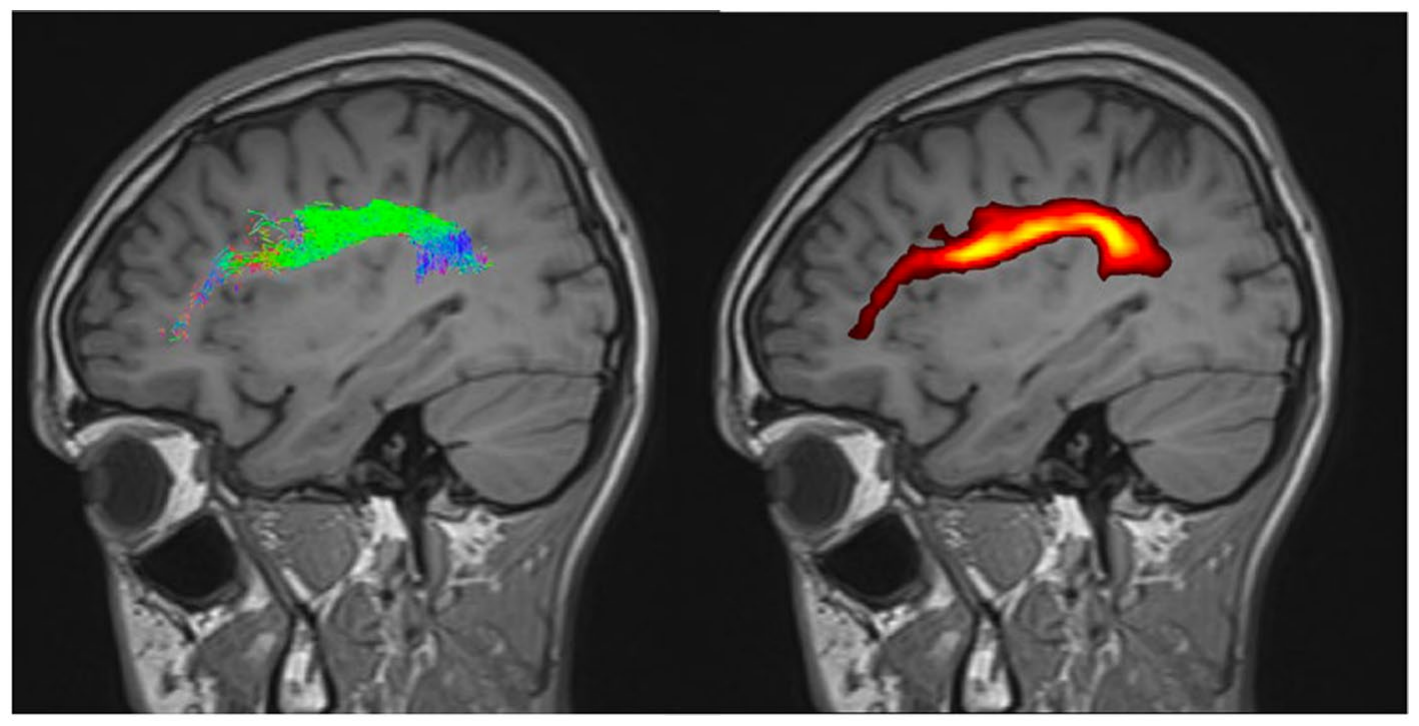
Illustration of streamline-based (left) and voxel-based (right) representations of the arcuate fasciculus in the same subject.

However, a key limitation of Tractfinder is that its outputs are generated in voxel (mask) space rather than as streamlines (.tck format). Voxel-based outputs represent a tract as a binary volume, whereas streamline-based representations model the tract as a collection of continuous fibre pathways (see Figure 1). Most clinicians and researchers are accustomed to working with streamline-based representations, which also underpins the majority of established tractography tools and visualisation workflows. This distinction has two practical consequences: direct comparison between Tractfinder outputs and conventional streamline-based tractography is not straightforward, and downstream analyses that rely on tractography frameworks are precluded, such as along-tract statistical profiling methods (e.g., Colby et al., 2012). Streamline-based outputs would therefore extend the compatibility and accessibility across both research and clinical contexts.

To address this limitation, we introduce Tractfinder-constrained Tractography (TcT), a streamline-based extension that constrains tractography to the binary maps produced by Tractfinder. Tractfinder-derived segmentations are used as binary mask regions-of-interest, restricting streamline propagation to within the tract volume and ensuring reconstructed streamlines remain consistent with Tractfinder outputs. TcT thereby generates streamline representations of the tracts while retaining the practical advantages that make Tractfinder clinically appealing. Importantly, as Tractfinder’s principal benefits are speed and convenience, TcT must preserve these characteristics to remain advantageous over conventional tractography approaches.

In this technical note, we present tract segmentations generated using TcT in a cohort of ten pre-operative paediatric tumour patients. Three white matter tracts were selected for evaluation – the corticospinal tract (CST), arcuate fasciculus (AF), and optic radiation (OR) – owing to their frequent involvement, displacement, or disruption in the context of paediatric supratentorial tumours, and their critical role in motor, language, and visual function, where surgical injury carries significant functional consequence. These results are compared with segmentations derived from conventional tractography methods. Comparisons are performed both quantitatively – assessing similarity between segmentation outputs using streamline-based bundle adjacency (Garyfallidis et al., 2012; Schilling et al., 2021) – and practically, evaluating workflow complexity and processing time. Through this analysis, we aim to determine whether TcT provides a streamlined, clinically focussed bridge between automated mask-based segmentation and streamline-based tractography.

## Methods

Following data acquisition and preprocessing, pre-operative diffusion MRI datasets were submitted to the TcT pipeline for automated delineation of three clinically relevant white matter tracts: the corticospinal tract (CST), arcuate fasciculus (AF), and optic radiation (OR). An equivalent processing stream using conventional tractography methods was applied to the same datasets to enable direct methodological comparison. Tract similarity between the two approaches was evaluated using the Bundle Adjacency (BA) metric, as implemented in the scilpy library (Renauld et al., 2026).

### Data collection

This study and the use of GOSH clinical data was approved by UCL REC (ID2780/005) and the UCL Institute of Child Health/GOSH joint R&D office (ref. 23NI01, IRAS project 332,150). All data were collected retrospectively from clinical neuroimaging acquired as part of routine pre-operative assessment at Great Ormond Street Hospital for Children, London. Informed consent was obtained in accordance with institutional requirements.

Diffusion MRI data were retrospectively collected from ten pre-operative paediatric patients with supratentorial tumours of varying histology; clinical diagnoses and surgical details are summarised in Table 1.

**Table 1:** Table summarising sample tumour types by tumour categories and hemisphere.

#	Pathology/Tumour	Hemisphere
1	Pleomorphic xanthoastrocytoma (PXA)	Right
2	Craniopharyngioma	Bilateral
3	Craniopharyngioma	Bilateral
4	Ependymoma	Left
5	Pilocytic astrocytoma	Right
6	Epidermoid cyst	Right
7	Glioneuronal tumour	Left
8	Low-grade glioma	Bilateral
9	Pleomorphic xanthoastrocytoma (PXA)	Left
10	Pilocytic astrocytoma	Right

Clinical neuroimaging data were acquired at Great Ormond Street Hospital, London (GOSH) using a Siemens MAGNETOM Prisma 3 T system. Structural T1-weighted images were obtained with the following parameters: TE/TR = 11/700 ms, with isotropic 1 mm^3^ voxels. Diffusion-weighted images were acquired using a multi-shell scheme comprising 60 directions at b = 1,000 s/mm^2^, 60 directions at b = 2,200 s/mm^2^, and 13 unweighted (b = 0) volumes, with TE/TR = 81/7300 ms and an isotropic voxel size of 2mm^3^, with a 0.2 mm slice gap.

### Data processing

All analyses were performed on a MacBook Air laptop (Apple Inc., Cupertino, CA, USA) with an Apple M2 chip and 16 GB RAM, running macOS Sequoia 15.1.1.

Image processing was performed using MRtrix3 (v3.0.4; https://www.mrtrix.org/) (Tournier et al., 2019). Preprocessing comprised the following steps: thermal noise removal (*dwidenoise*) (Veraart et al., 2016), Gibbs ringing correction (*mrdegibbs*) (Kellner et al., 2016), motion and distortion correction via FSL’s *eddy* (*dwifslpreproc*) (Andersson and Sotiropoulos, 2016), and bias field correction using the ANTs algorithm (*dwibiascorrect*) (Smith et al., 2004; Zhang et al., 2001), followed by brain mask estimation. Diffusion data were linearly registered to MNI152 standard space (Fonov et al., 2011) using FSL’s *FLIRT* (v6.0) (Jenkinson et al., 2002, 2012). Affine rather than non-linear registration was employed to account for individual global anatomical variability while limiting streamline distortions caused by local registration errors. Affine registration also offers advantages in terms of processing speed and reproducibility, with non-linear algorithms more likely to require parameter adjustment or manual intervention. Fibre orientation distributions (FOD) were estimated using multi-shell multi-tissue constrained spherical deconvolution (MSMT-CSD) (Jeurissen et al., 2014), with tissue response functions derived using the *Dhollander* unsupervised algorithm (Dhollander et al., 2016).

When required, tumour masks were estimated using the nnU-Net-based segmentation framework of Ruffle et al. (2023), which provides automated brain tumour delineation from structural MRI and has been validated for use with routinely acquired, heterogeneous clinical data. Critically, the framework was trained on incomplete imaging datasets and maintains robust segmentation performance in the absence of a full multimodal protocol; lesion masks were therefore derived from T1-weighted images alone, reducing both data requirements and processing time.

### Tractfinder-constrained tractography pipeline

Tractfinder was applied to generate probability maps for three bilateral white matter tracts: the CST, AF, and OR. Tractfinder generates pseudo-probability maps of tract location – voxel-wise inner products of a registered orientation atlas and subject FOD images, expressed in arbitrary and dimensionless units (Young et al., 2024). Where tumour mass effect resulted in tract displacement, a tumour deformation model was applied incorporating the lesion mask (Young et al., 2022). Each dataset was visually inspected to determine whether deformation modelling was required and to verify the appropriateness of the *k* parameter selected. Manual adjustment of the deformation parameter (*k*) was required in only one case, suggesting the pipeline is largely robust to automation, though visual quality control remains advisable. Tractfinder probability maps were thresholded at 0.05, a value empirically determined to be suitable for converting pseudo-probability maps to binary segmentation in the original Tractfinder methodology (Young et al., 2024). This was computed using *mrthreshold* as implemented in MRtrix3.

These binary masks were subsequently used to constrain targeted probabilistic streamline tractography, performed using the *iFOD2* algorithm (Tournier et al., 2010), with the *-mask* option applied to restrict streamline propagation to the Tractfinder-defined tract volume. Tractography was run with high-density seeding (*−seeds 1,000,000*) with no fixed streamline count (*−select 0*). MRTrix3 default settings were used for step size (0.5 x voxel size), angle threshold (45), FOD cutoff (0.1), and minimum/maximum streamline length (5 x voxel size / 100 x voxel size). Final tractograms were resampled to 2000 streamlines per tract using *tckedit* for visualisation.

### Conventional tractography pipeline

For comparison, targeted probabilistic streamline tractography was performed using the iFOD2 algorithm (Tournier et al., 2010), seeded from manually placed regions of interest (ROIs) defined following the protocol described in Young et al. (2024, Supplementrary Material D1). These are summarised in Table 2.

**Table 2:** Regions of interest (ROIs) used for conventional tractography, following the protocol of Young et al. (2024).

Tract	Seed	Include	Exclude
AF	White matter medial to angular gyrus (coronal plane; “green triangle” on colour FA maps)	Descending section of AF (axial plane)	Midline, superior fronto-occipital fasciculus, ipsilateral cerebral peduncles, sagittal stratum, corona radiata, external capsules
CST	Posterior limb of internal capsule (3 consecutive axial slices)	Cerebral peduncles, CST in mid-pons	Cerebellar peduncles (coronal slice), medial lemniscus (axial slice), midline, superior fronto-occipital fasciculus
OR	Lateral geniculate nucleus (axial plane)	Sagittal stratum (coronal plane)	Anterior & inferior to most anterior point of lateral ventricles, level of superior reach of lateral ventricles, splenium of corpus callosum, fornix

AF, Arcuate Fasciculus; CST, Corticospinal Tract; OR, Optic Radiation.

Tractography was initialised from FODs derived from MSMT-CSD (Jeurissen et al., 2014), incorporating white matter and grey matter tissue compartments. Tractography was run with high-density seeding (*−seeds 1,000,000*) from manually-placed ROIs, with no fixed streamline count (*−select 0*). MRTrix3 default settings were used for step size, angle threshold, FOD cutoff, and minimum/maximum streamline length. Final tractograms were resampled to 2000 streamlines per tract using *tckedit* for visualisation.

### Quantitative comparisons

Conventional tractography streamlines were post-processed to remove spurious streamlines using a track density imaging (TDI) approach. A TDI map was generated using *tckmap*, and a binary mask was created by thresholding at a minimum of 5 streamlines per voxel. Streamlines were then filtered using *tckedit* with the TDI-derived mask, rejecting any streamline that did not pass through at least one voxel meeting the minimum density threshold of 5 streamlines per voxel.

Quantitative comparisons between the Tractfinder-constrained and conventional tractography outputs were performed using BA, as implemented in the scilpy tractography package (Garyfallidis et al., 2012; Schilling et al., 2021).

## Results

### Quantitative comparisons

**Figure 2:**
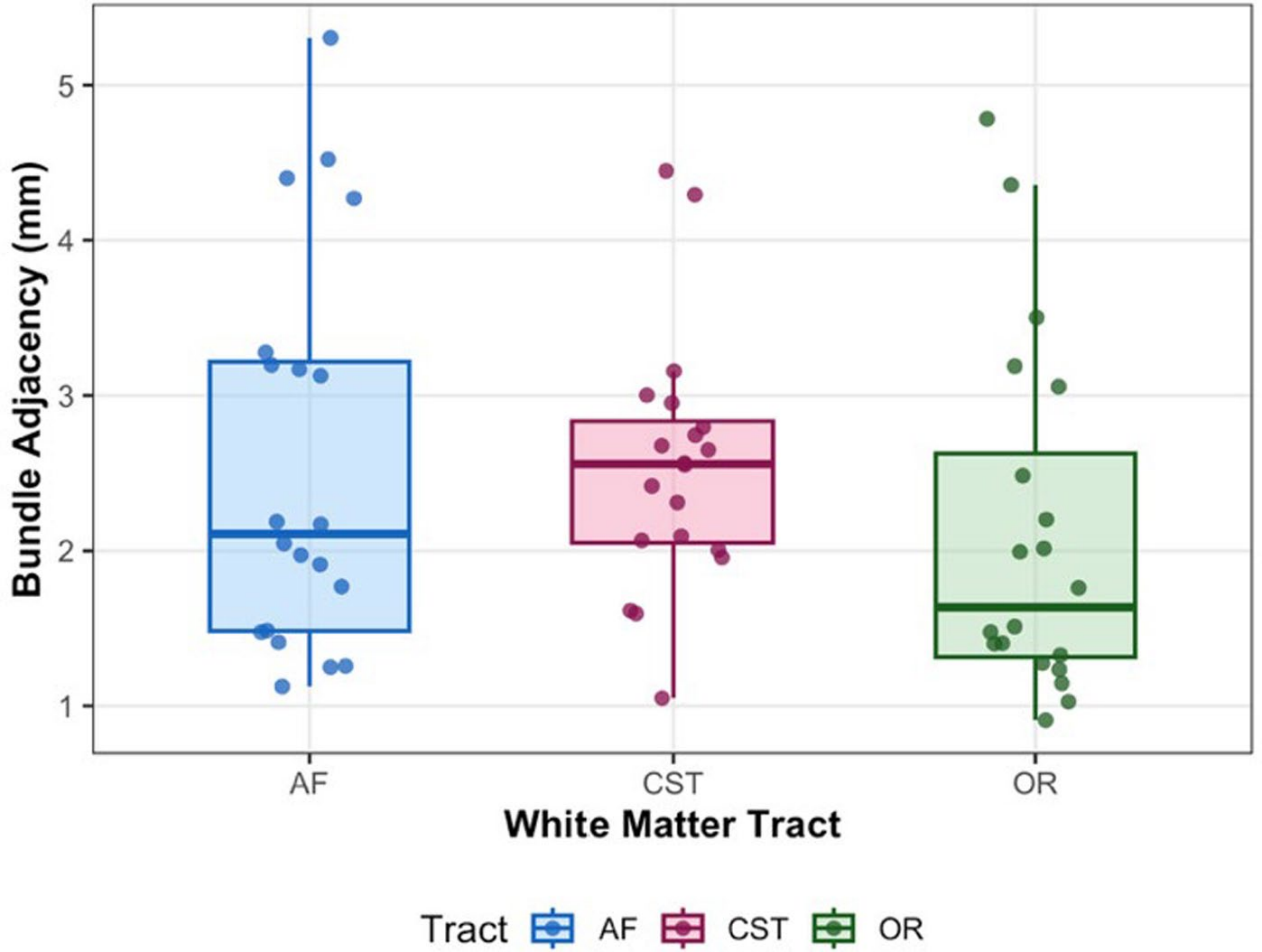
Box-and-whisker plots of BA scores comparing tract segmentations generated by the Tractfinder-constrained Tractography (TcT) and conventional tractography pipelines across three white matter tracts: the corticospinal tract (CST), arcuate fasciculus (AF), and optic radiation (OR).

Tract overlap between the Tractfinder-constrained and conventional tractography pipelines was generally good. Mean BA scores compared favourably against published benchmarks comparing inter-protocol conventional probability tractography pipelines, where mean BA has been reported at 4.3 mm (Schilling et al., 2021); per-tract averages are presented in Table 3 and full results in Figure 2.

**Table 3:** Mean streamline-based BA scores quantifying spatial correspondence between Tractfinder-constrained tractography (TcT) and conventional tractography outputs.

Tract	Bundle adjacency, mean (StDev)
Arcuate fasciculus	2.57 mm (1.27)
Cortico-spinal tract	2.55 mm (0.82)
Optic radiation	2.10 mm (1.12)

**Figure 3:**
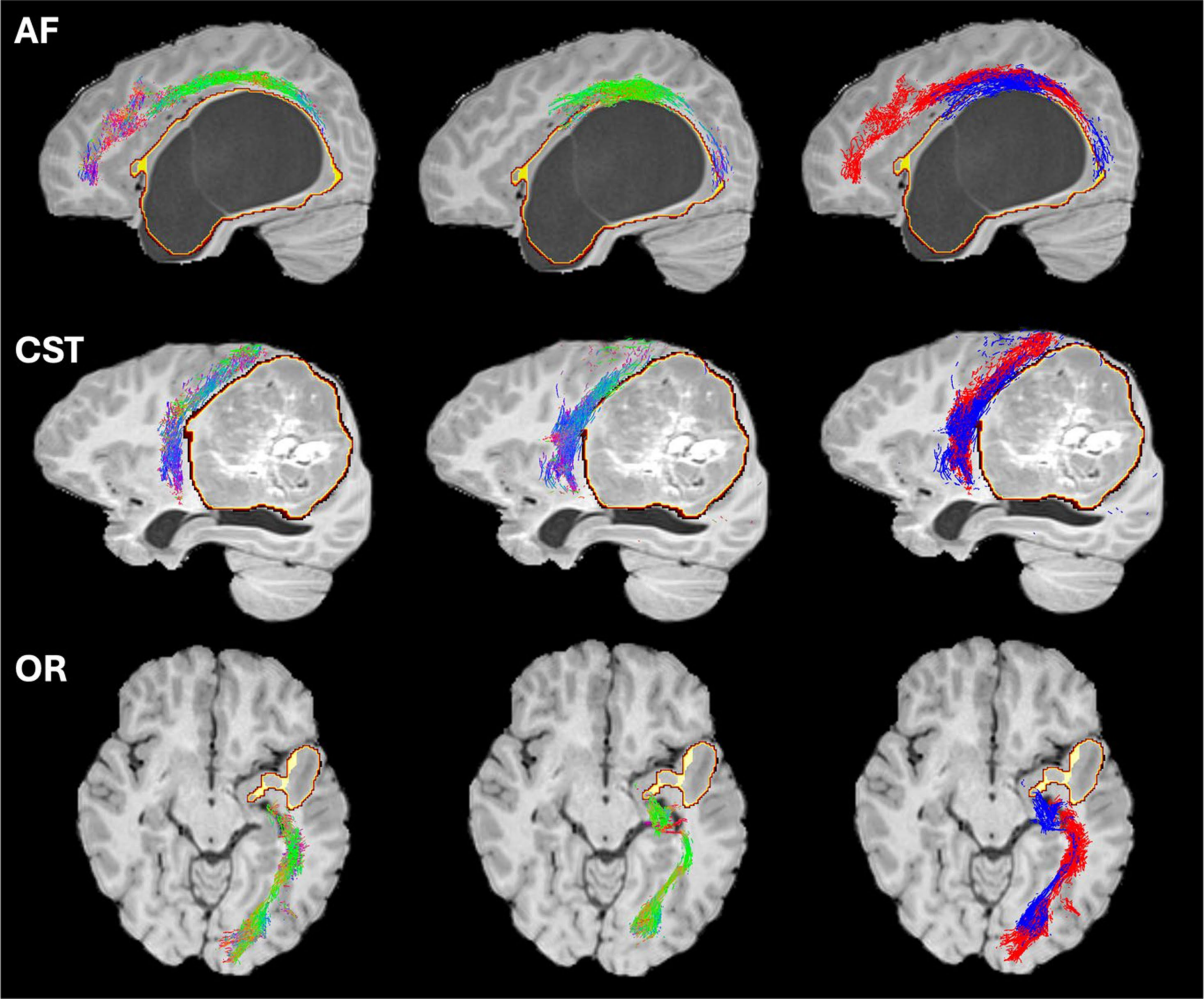
Side-by-side visualisations of Tractfinder-constrained tractography (TcT; left column) and conventional tractography (middle column) outputs for the right arcuate fasciculus (AF), left corticospinal tract (CST), and left optic radiation (OR), shown in patients aged 10, 15, and 12 years respectively, selected to illustrate cases in which tumour location or size notably influenced tract morphology. The right column displays overlapping outputs: TcT in red, Tractography in blue.

Figure 3 displays representative examples comparing TcT and conventional tractography reconstructions of the CST, AF, and OR in cases where tract morphology was influenced by tumour location or size. These cases illustrate the challenges posed by peritumoral displacement and disruption for automated tract delineation. Bundle Adjacency scores for the illustrated cases were: AF = 4.5 mm, CST = 2.1 mm, and OR = 3.1 mm.

### Practical considerations

A qualitative and practical comparison of the two pipelines is summarised in Table 4. The TcT pipeline was substantially faster, requiring approximately 5–15 min per subject (excluding preprocessing and tumour segmentation); the nnU-Net-based tumour segmentation step adds minimal overhead, with processing times of approximately 10–15 s per patient on GPU-accelerated hardware, though this will vary depending on available computational resources (Ruffle et al., 2023). Comparatively, conventional tractography took 1–2 h to complete (excluding preprocessing), and required manual ROI placements, representing a considerable increase in operator burden.

**Table 4:** Comparison of Tractfinder-constrained (TcT) and conventional tractography pipelines across key methodological dimensions.

Feature	Tractfinder-constrained tractography (TcT)	Conventional tractography
Automation	Fully automated (single script)	Semi-manual (requires manual ROI placement)
Preprocessing	Standard DWI preprocessing + tumour/lesion mask	Standard DWI preprocessing
Tract delineation	Automated via Tractfinder probability maps	Manual ROI placement per tract
Tractography	iFOD2, constrained by Tractfinder binary mask	iFOD2, seeded from manual ROIs
User input required	Lesion mask; optional *k* parameter adjustment (in the presence of significant abnormalities)	Seed, inclusion & exclusion ROIs for each tract
Operator experience required	Minimal	Neuroanatomical expertise required
Processing time (per subject, excluding preprocessing)	~5–15 min	~1–2 h
Scalability	High, suitable for large cohorts	Limited by manual processing burden

## Discussion

This technical note introduces Tractfinder-constrained Tractography (TcT) as a methodological extension of Tractfinder (Young et al., 2022, 2024), enabling the generation of streamline-based white matter tract reconstructions within a fully automated, clinically focussed pipeline. The principal finding is that TcT produces tract segmentations with good spatial agreement to, and in the same form as, those derived from conventional tractography, while offering substantially reduced processing time and operator burden. It should be noted, however, that TcT is benchmarked against conventional tractography rather than an independent ground truth, and findings should therefore be interpreted as agreement rather than validation of anatomical accuracy.

Mean BA scores across all three tracts (CST: 2.5 mm, AF: 2.6 mm, OR: 2.1 mm) compared favourably against published inter-protocol benchmarks for probabilistic tractography, where median BA between different traditional pipelines has been reported at 4.3 mm (Schilling et al., 2021). Notably, TcT–conventional tractography BA scores are more comparable to within-protocol variability (~2 mm). These results hold across a clinically heterogeneous cohort encompassing a range of tumour types, locations, and degrees of tract displacement, lending confidence to the generalisability of the approach.

A key practical advantage of TcT is its accessibility. The entire pipeline runs as a single automated script on a standard MacBook Air laptop, requiring no dedicated GPU infrastructure or case-by-case manual ROI placement, though neuroanatomical knowledge remains important for appropriate interpretation and quality control of outputs. This contrasts markedly with conventional tractography, which requires manual placement of seed, inclusion, and exclusion ROIs for each tract (a time-consuming process demanding considerable operator experience and introducing inter-rater variability), tractography execution, and operator quality control. Despite the variability in conventional tractography processing times across users, departments, and practices, TcT consistently demonstrated substantially reduced processing times, representing a meaningful step towards scalable tract delineation in clinical and research settings, pending further validation in larger and more diverse cohorts. This may be particularly advantageous in time-critical scenarios such as intraoperative MRI, where rapid tract delineation is essential for surgical decision-making in cases where tumours are in close proximity to eloquent white matter pathways. Finally, the conversion of Tractfinder outputs from voxel-space masks to streamline representations substantially extends analytical compatibility. Streamline-based outputs enable downstream analyses within other tractography-dependent frameworks, further broadening the utility of Tractfinder.

Several limitations of current methods should be acknowledged. While BA captures spatial proximity, it does not quantify topological correspondence, and future evaluation should incorporate complementary metrics selected according to the specific question being addressed. The cohort is also small (*n* = 10) and comprises predominantly low-grade tumours. While this reflects the typical distribution of paediatric supratentorial tumours, it limits generalisability to tumour types with a greater effect on white matter structure, such as intra-axial gliomas, which grow within and infiltrate white matter, and high-grade lesions associated with more extensive peritumoral oedema, where additional challenges for automated tract delineation may arise. Additionally, ground truth tract anatomy is unavailable in this population – precluding absolute validation – and comparison against conventional tractography serves as a surrogate measure of agreement rather than accuracy. A further consideration is the dependency between TcT outputs and Tractfinder probability maps: because TcT streamlines are restricted to regions defined by atlas-derived priors, tract geometry will inevitably reflect these constraints to some degree, and the observed agreement with conventional tractography may partly capture shared anatomical assumptions rather than fully independent corroboration from diffusion data alone. This is an inherent feature of constrained tractography approaches, but has important implications in the context of paediatric tumours where anatomy may be severely distorted. While the incorporation of a lesion mask partially mitigates this, cases with highly infiltrative margins may still pose a challenge for atlas-guided delineation. Indeed, in a small number of cases, manual adjustment of the deformation model parameter (*k*) was required, indicating that full automation may not always be achieved without some degree of visual quality control. Finally, although the threshold of 0.05 was applied in accordance with the original Tractfinder methodology (Young et al., 2024), this represents a parameter worth exploring in future work, particularly in cases with significant tumour-related distortion. The current technical note introduces TcT and demonstrates its application in a representative clinical cohort, with more comprehensive evaluation across a wider range of tumour types anticipated in future work. While commercial platforms for surgical planning increasingly offer rapid multi-shell CSD-based probabilistic tractography (Ashmore et al., 2020), TcT’s atlas-guided anatomical constraints offer tract delineation in the presence of tumour-related distortion, without the need for manually placed ROIs. Future work should evaluate TcT against these and other established methods, as well as in larger, multicentre cohorts and explore its integration into prospective surgical planning workflows. More broadly, Tractfinder should be trialled on other pathologies, healthy populations, and non-clinical research contexts in which tract morphology and image quality may differ substantially from those represented here. Future work could also characterise tract involvement relative to lesion location, to better understand tractography performance as a function of proximity to pathology. Extension to additional clinically relevant tracts, and formal assessment of inter-rater and inter-site reproducibility, would further consolidate its utility as a standardised tract delineation tool.

## Data Availability

Scripts for running Tractfinder and generating custom tract atlases are available at: https://github.com/tractfinder/tractfinder. Tract orientation atlases (and corresponding training streamlines) for the AF, CST, and OR are openly available for non-commercial use: https://doi.org/10.5281/zenodo.10149873. The clinical neuroimaging data from Great Ormond Street Hospital cannot be publicly shared to maintain patient confidentiality. Requests to access these datasets should be directed to danakanel@gmail.com.
